# Assessment of the Prognostic Value of Two Common Variants of BRCA1 and BRCA2 Genes in Ovarian Cancer Patients Treated with Cisplatin and Paclitaxel: A Gynecologic Oncology Group Study

**DOI:** 10.3389/fonc.2013.00206

**Published:** 2013-08-12

**Authors:** Chunqiao Q. Tian, Kathleen M. Darcy, Thomas C. Krivak, Julie A. DeLoia, Deborah Armstrong, Warren Davis, Hua Zhao, Kirsten Moysich, Christine B. Ambrosone

**Affiliations:** ^1^Gynecologic Oncology Group Statistical and Data Center, Buffalom, NY, USA; ^2^Magee Womens Hospital, University of Pittsburgh, Pittsburgh, PA, USA; ^3^Georgetown University, Washington, DC, USA; ^4^John Hopkins Kimmel Cancer Center, Baltimore, MD, USA; ^5^Department of Cancer Prevention and Control, Roswell Park Cancer Institute, Buffalo, NY, USA

**Keywords:** BRCA, polymorphism, ovarian cancer, chemotherapy, prognosis

## Abstract

**Purpose:** BRCA1/BRCA2 germline mutations appear to enhance the platinum-sensitivity, but little is known about the prognostic relevance of polymorphisms in BRCA1/BRCA2 in epithelial ovarian cancer (EOC). This study evaluated whether common variants of BRCA1/BRCA2 are associated with progression-free survival (PFS) and overall survival (OS) in patients with advanced stage sporadic EOC.

**Experimental Design:** The allelic frequency of BRCA1 (2612C > T, P871L-rs799917) and BRCA2 (114A > C, N372H-rs144848) were determined in normal blood DNA from women in Gynecologic Oncology Group protocol #172 phase III trial with optimally resected stage III EOC treated with intraperitoneal or intravenous cisplatin and paclitaxel (C + P). Associations between polymorphisms and PFS or OS were assessed.

**Results:** Two hundred and thirty-two women were included for analyses. African Americans (AA) had different distributions for the two polymorphisms from Caucasians and others. For non-AA patients, the genotype for BRCA1 P871L was distributed as 38% for CC, 49% for CT, and 13% for TT. Median PFS was estimated to be 31, 21, and 21 months, respectively. After adjusting for cell type, residual disease, and chemotherapy regimen, CT/TT genotypes were associated with a 1.40-fold increased risk of disease progression [95% confidence interval (CI) = 1.00–1.95, *p* = 0.049]. After removing seven patients with known BRCA1 germline mutations, the hazard ratio (HR) was 1.36 (95% CI = 0.97–1.91, *p* = 0.073). The association between BRCA1 P871L and OS was not significant (HR = 1.25, 95% CI = 0.88–1.76, *p* = 0.212). Genotype distribution of BRCA2 N372H among non-AA patients was 50, 44, and 6% for AA, AC, and CC, respectively and there is no evidence that this BRCA2 polymorphism was related to PFS or OS.

**Conclusion:** Polymorphisms in BRCA1 P871L or in BRCA2 N372H were not associated with either PFS or OS in women with optimally resected, stage III EOC treated with cisplatin and paclitaxel.

## Introduction

The standard treatment for advanced epithelial ovarian cancer (EOC) begins with cytoreductive surgery followed by chemotherapy consisting of platinum and taxane (1–3). Although tumor response rate to this regimen is as high as 70–80%, the majority of patients relapse within 2–3 years (3, 4). Identification of biomarkers that predict resistance to platinum/taxane-based chemotherapy may allow alternative therapeutic options to be considered for this high-risk group (5).

Most ovarian cancers are due to sporadic events and 8–10% are attributable primarily to germline mutations in breast cancer 1 (BRCA1) or BRCA2 genes (6). BRCA1 and BRCA2 are considered tumor suppressor genes, and loss-of-function mutations confer a significantly increased risk of developing breast and ovarian cancer (6). BRCA-associated ovarian cancer patients appear to display a better response to platinum agents as compared to sporadic patients (7–11), likely due to a BRCA-deficient tumor having suboptimal DNA repair and enhanced sensitivity to DNA-damaging drugs (12–14). In sporadic EOC, the inactivation of BRCA through various mechanisms is common and studies in this setting have demonstrated that low levels of BRCA1 mRNA or protein predict improved outcome following platinum-based chemotherapy (15–18). Associations between BRCA1 and treatment outcome have been reported in several solid tumors including lung and colorectal cancers (19–21). Taken together, BRCA1 and BRCA2 appear to play critical roles in development of ovarian cancer and modulation of chemotherapy responsiveness.

A number of polymorphisms have been identified in the BRCA1 gene and P871L (rs799917) is one of the most common variants with a minor allele (T) frequency of 32% among Caucasian cancer patients (22, 23). In the BRCA2 gene, N372H (rs144848) which results in an amino acid change is the most common polymorphism with a minor allele (C) frequency of 27% among Caucasian cancer patients (22). It is unclear if these common variants in BRCA1 or BRCA2 genes impact therapeutic efficacy and clinical outcome. The prognostic value of these two most common variants in the BRCA1 and BRCA2 genes was examined in normal DNA from women who participated in Gynecologic Oncology Group (GOG) protocol #172, a phase III trial where patients with stage III EOC were treated with cisplatin + paclitaxel (C + P).

## Materials and Methods

### Study population

Patients in this study participated in a randomized phase III trial, GOG#172, and had normal DNA available for genotyping. Details regarding eligibility criteria, treatment, and end points, and the survival advantage for women randomized to intraperitoneal C + P compared with intravenous C + P for GOG#172 have been reported elsewhere (28). Women on this study provided written informed consent to participate in GOG#172 and provide a blood specimen for research consistent with all federal, state, and local requirements.

### Genotyping

Genomic DNA was extracted from white blood cells recovered from whole blood as described previously (29). BRCA1 P871L (rs799917) and BRCA2 N372H (rs144848) polymorphisms were genotyped using a MALDI-TOF iPLEXTMGOLD assay (Sequenom, San Diego, CA, USA).

### Statistical analysis

The genotype data were tested for Hardy–Weinberg equilibrium (HWE) using exact permutation test. Associations between polymorphisms and clinical characteristics were assessed using Wilcoxon rank-sum for continuous variables and using Pearson-χ^2^ or Fisher exact test for categorical variables. Kaplan–Meier procedure was used to estimate progression-free survival (PFS) and overall survival (OS) by genotype. Hazard ratios were estimated using a Cox regression model adjusted for prognostic factors including cell type (clear cell/mucinous vs. other histologic subtypes), residual disease status (gross vs. no gross), and treatment regimen (intraperitoneal vs. intravenous). These variables were selected for adjustment based on previous GOG studies (28–30). Previous studies demonstrated that the distributions of polymorphisms in BRCA1 and BRCA2 varied by race (22), analyses of PFS/OS were performed without African American (AA) women (data shown) and with AA women (data not shown). As some women in GOG#172 had a known germline mutation in the BRCA 1 gene, PFS/OS analyses were performed with and without these patients.

## Results

Patient characteristics for the 232 women in this study are shown in Table 1 and are representative of that observed in the entire GOG#172 protocol (28). Approximately 55% of the women had intravenous (IV) C + P, with the remainder receiving IP therapy. At the time of analysis, 148 patients had died, 55 women were alive with no evidence of disease, and 29 were alive with documented recurrence/disease progression. The median follow-up period for those still alive was 87 months. Overall, the median PFS of this population was 21.8 months and the median OS was 57.8 months.

**Table 1:** **Clinical Characteristics (*N* = 232)**.

	No. (%)
Age (years)
<55	104 (44.8)
55–64	63 (27.2)
≥65	65 (28.0)
Median (range)	57 (32–83)
Race
White	213 (91.8)
Black	7 (3.0)
Other	12 (5.2)
GOG performance status
0	101 (43.5)
1	115 (49.6)
2	16 (6.9)
Cell type
Serous	178 (76.7)
Endometrioid	15 (6.5)
Clear cell (CC)	14 (6.0)
Mutinous (MU)	1 (0.4)
Other^1^	24 (10.3)
Tumor grade
1	23 (9.9)
2	92 (39.7)
3 or clear cell	117 (50.4)
Histology
HGS^2^	157 (67.7)
Non-HGS	75 (32.3)
Residual disease
Microscopic	97 (41.8)
Gross	135 (58.2)
Treatment^3^
IP Cis + P	105 (45.3)
IV Cis + P	127 (54.7)

*1. Other histologic subtypes included: mixed epithelial, undifferentiated, transitional cell, and adenocarcinoma, not otherwise specified*.

*2. HGS: high-grade (grade 2 or grade 3) serous tumor*.

*3. IP, intraperitoneal; IV, Intravenous; Cis, cisplatin; P, paclitaxel*.

### BRCA1 P871L polymorphism (*N* = 232)

**Figure 1:**
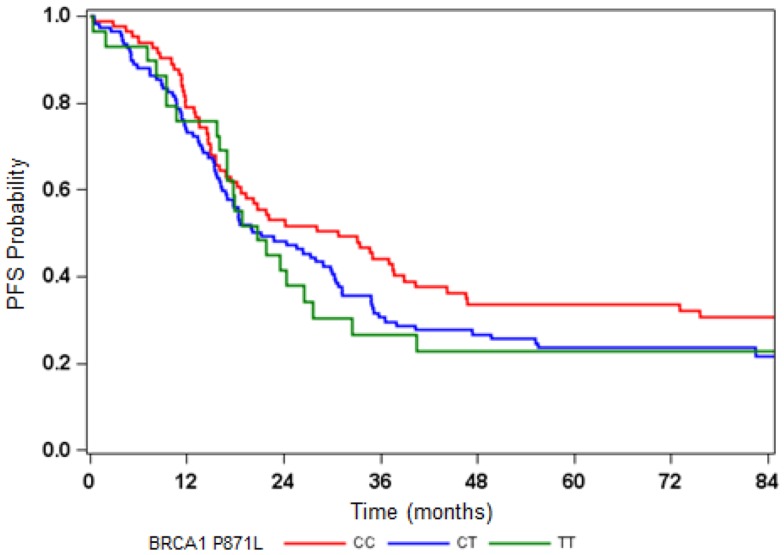
**Disease progression-free survival (PFS) by BRCA1P871L polymorphism**.

**Figure 2:**
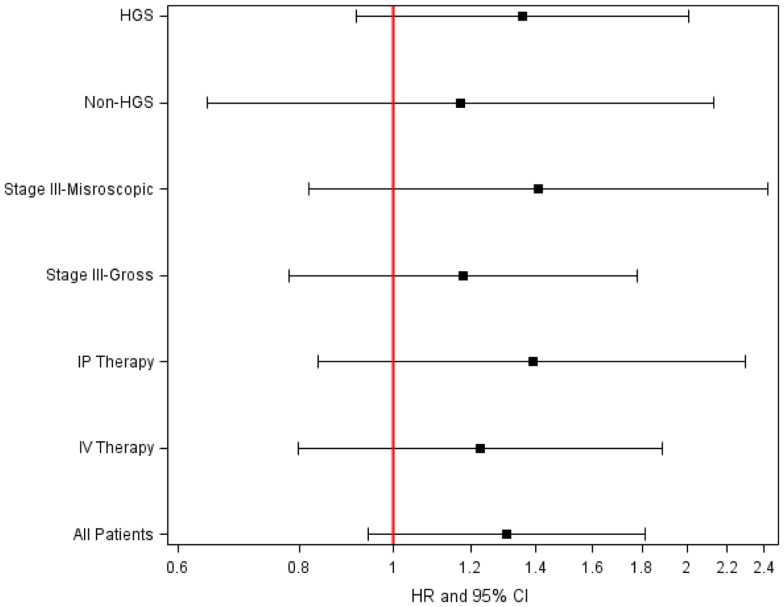
**Adjusted hazard ratio (HR) of disease progression for CT/TT vs. CC genotype in BRCA1P87 in women with optimally resected stage III epithelial ovarian cancer stratified by treatment or residual disease**.

The BRCA1 P871L polymorphism was associated with race (*p* < 0.001) but not with other clinical variables (Table 2). In particular, there was no difference in genotype distribution between HGS and non-HGS tumors (*p* = 0.700). All seven AA women exhibited the TT genotype. Among non-AA patients, the genotype for P871L in BRCA1 was distributed as 38% for CC, 49% for CT, and 13% for TT, consistent with HWE (*p* = 0.569). Median PFS was 31, 21, and 21 months, respectively (log-rank test for CC vs. CT/TT: *p* = 0.109, Figure 1). Median OS was 70, 55, and 59 months for CC, CT, and TT genotypes, respectively (log-rank test for CC vs. CT/TT: *p* = 0.359). After adjusting for cell type, residual disease, and chemotherapy regimen, patients with CT/TT genotypes vs. the CC genotype had an increased risk of disease progression (HR = 1.40, 95% CI = 1.00–1.95, *p* = 0.049, Table 2), and a similar risk of death (HR = 1.25, 95% CI = 0.88–1.76, *p* = 0.212, Table 3). When the seven women with a known BRCA1 mutation were removed from the analysis, the association between the BRCA1 P871L polymorphism and PFS (HR = 1.36, 95% CI = 0.97–1.91, *p* = 0.073) was no longer statistically significant (Table 3). Subgroup analyses stratified by histology (HGS vs. non-HGS), disease residual (microscopic vs. gross) or treatment arm (IV vs. IP) illustrated trends suggestive of a modest elevation in risk of disease progression for women with the CT/TT vs. CC genotype in these subgroups, but these associations were not statistically significant (Figure 2).

**Table 2:** **Associations between the BRCA1 P871L or BRCA2 N372H Polymorphism and Clinical Characteristics**.

	BRCA1 P871L				BRCA2 N372H^1^			
	CC	CT	TT		AA	AC	CC	
	No. (%)	No. (%)	No. (%)	*p*-Value	No. (%)	No. (%)	No. (%)	*p*-Value
Age in years (median)	56.9	56.4	55.3	0.369^a^	54.9	57.9	55.6	0.820^a^
Race
White	81 (38.0)	104 (48.8)	28 (13.2)	<0.001^c^	106 (50.5)	91 (43.3)	13 (6.2)	0.155^c^
Black	0 (0)	0 (0)	7 (100)		6 (100)	0 (0)	0 (0)	
Other	4 (33.3)	7 (58.3)	1 (8.3)		5 (41.7)	6 (50.0)	1 (8.3)	
Performance status
0	34 (33.7)	53 (52.5)	14 (13.9)	0.461^b^	51 (51.0)	44 (44.0)	5 (5.0)	0.791^b^
1 or 2	51 (38.9)	58 (44.3)	22 (16.8)		66 (51.6)	53 (41.4)	9 (7.0)	
Histology
HGS^2^	55 (35.0)	76 (48.4)	26 (16.6)	0.700^b^	79 (51.3)	66 (42.9)	9 (5.8)	0.961^b^
Non-HGS	30 (40.0)	35 (46.7)	10 (13.3)		38 (51.4)	31 (41.9)	5 (6.8)	
Tumor residual
No gross	35 (36.1)	46 (47.4)	16 (16.5)	0.940^b^	48 (50.0)	42 (43.8)	6 (6.3)	0.944^b^
Gross	50 (37.0)	65 (48.2)	20 (14.8)		69 (52.3)	55 (41.7)	8 (6.1)	
Treatment^3^
IP C + P	37 (35.2)	51 (48.6)	17 (16.2)	0.914^b^	52 (50.0)	46 (44.2)	6 (5.8)	0.889^b^
IV C + P	48 (37.8)	60 (47.2)	19 (15.0)		65 (52.4)	51 (41.1)	8 (6.5)	

*^1^Genotype in BRCA2 N372H not determined for four specimens; CC, clear cell; MU, mucinous*.

*^2^HGS, high-grade (grade 2 or grade 3) serous tumor*.

*^3^IP, intraperitoneal; IV, intravenous; C, cisplatin; P, paclitaxel*.

*^a^Wilcoxon rank-sum test*.

*^b^Pearson-χ^2^ test*.

*^c^Fisher exact test*.

**Table 3:** **Progression-free survival (PFS) and overall survival (OS) by BRCA1 P871L and BRCA2 N372H polymorphisms for patients treated with cisplatin/paclitaxel-based chemotherapy**.*

	No. (%)	PFS			OS		
		HR	95% CI	*p-*Value	HR	95% CI	*p*-Value
**BRCA1 P871L**							
All patients							
CC	85 (37.8)	Referent			Referent		
CT	111 (49.3)	1.37	0.96–1.94	0.079	1.21	0.84–1.74	0.300
TT	29 (12.9)	1.53	0.93–2.51	0.092	1.39	0.82–2.33	0.220
CC + TT		1.40	1.00–1.95	0.049	1.25	0.88–1.76	0.212
By excluding seven cases with known brca1 mutations							
CC	83 (38.1)	Reference			Reference		
CT	108 (49.5)	1.32	0.93–1.88	0.123	1.21	0.84–1.76	0.304
TT	27 (12.4)	1.54	0.93–2.55	0.092	1.31	0.76–2.26	0.327
CC + TT		1.36	0.97–1.91	0.073	1.23	0.87–1.76	0.244
**BRCA2 N372H**							
AA	117 (51.3)	Referent			Referent		
AC	97 (42.5)	1.08	0.78–1.50	0.659	1.20	0.85–1.69	0.311
CC	14 (6.1)	0.81	0.42–1.57	0.533	0.76	0.36–1.59	0.462
AC + CC		1.03	0.75–1.42	0.844	1.13	0.80–1.58	0.492

*Hazard ratio (HR) and 95% confidence interval (CI) estimated from Cox regression model adjusted for cell type, tumor residual, and type of treatment*.

*Analysis limited to patients by excluding African American women.

### BRCA2 N372H polymorphism (*N* = 228)

**Figure 3:**
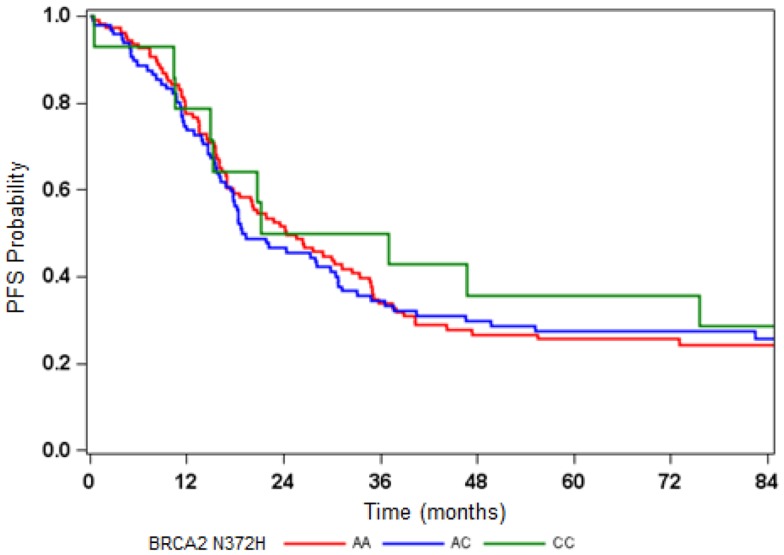
**Disease progression-free survival (PFS) by BRCA2 N372H polymorphism**.

All six AA women exhibited the CC genotype in BRCA2 N372H (Table 2) but the relationship between the N372H polymorphism and race did not reach statistical significance in this study. Among the non-AA patients, the genotype for this polymorphism was distributed as 50% for AA, 44% for AC, and 6% for CC (*p* = 0.381 for HWE) (Table 2). There was no evidence that the BRCA2 N372H polymorphism was associated with PFS or OS (Figure 3; Table 2).

## Discussion

Both preclinical and clinical data suggest that alterations in BRCA1 or BRCA2 have prognostic value in ovarian cancer. Patients with germline mutations have improved clinical outcomes following the platinum chemotherapy (9–11, 31, 37). Increased sensitivity to DNA-damaging anti-cancer drugs has been associated with BRCA1 or BRCA2 functional loss involving germline mutations or epigenetic changes. To extend these prior studies, we evaluated the prognostic value of the two common polymorphisms in BRCA1 and BRCA2 in GOG#172. Common polymorphisms in BRCA1 P871L and BRCA2 N372H polymorphism were not strongly associated with clinical outcome (PFS or OS) in patients treated with C + P. Patients with the CT or TT vs. the CC genotype in BRCA1 P871L had a modest increased risk of disease recurrence attributable at least in part to the subset of GOG#172 patients with a known germline mutation in BRCA1.

Several factors prompted our interest in the BRCA1 P871L and the BRCA2 N372H polymorphisms. First, both of these polymorphisms are very common and if one of them was found to be strongly associated with PFS or OS that biomarker would be able to be reliably detected in a minimally invasive blood sample. Second, relatively little is known about the biological, functional, and clinical impact of these polymorphisms. For example, the functional consequence of C → T transition in P871L in BRCA1 is not well understood. It is hypothesized that a C → T transition in this polymorphism alters the expression and/or function of BRCA1 enhancing chemosensitivity and outcome similar to that reported for ERCC1 (29, 34– 36). The functional effects of BRCA1 P871L have been evaluated in breast cancer cell lines, and the different genotypes had distinctly different levels of BRCA1 protein (39). Based on this finding we expected enhanced sensitivity to platinum in women with the BRCA1 polymorphism. Several studies examined the association between BRCA1 P871L polymorphism and risk of developing ovarian. While there were reports that the P871L polymorphisms was associated with an increased risk of developing ovarian cancer (23, 24), this correlation was not confirmed based on large-scale studies (25, 26). In contrast, a recent study reported that this polymorphism (P871L) was associated with a reduced risk of developing cervical cancer (32). Among advanced gastric cancer patients treated with C + P, the P871L polymorphism was associated with improved PFS and OS (33).

Third, BRCA1 may have different roles in modulation of responses to DNA-damaging drugs compared with anti-microtubule drugs. It is proposed that BRCA1 mediates resistance to platinum agents involving activation of NER, HR, and FA/BRCA repair pathways and sensitivity to taxane-based chemotherapy involving mitotic spindle arrest followed by JNK/SAPK mediated apoptosis (16, 31, 37). Thus far most clinical studies have focused on BRCA1 deficiency as a potential biomarker of response to platinum chemotherapy but not as a marker of taxane resistance. The potential contradictory effects of BRCA1 on the two most common agents used to treat advanced ovarian cancer make it difficult to appreciate how alterations in BRCA1 mediate tumor response in patients treated with a combination of C + P.

Recent studies on BRCA1/BRCA2 have concluded that a germline mutation in BRCA1 or BRCA2 was associated with improved survival in ovarian cancer patients and BRCA2 carriers had the best prognosis (40–42). Some investigators have proposed that BRCA1 and BRCA2 work at different stage in DNA damage response and in DNA repair. BRCA1 is functional in both checkpoint activation and DNA repair, whereas BRCA2 is a mediator of the core mechanism of homologous recombination (HR) (43). In the present study, we evaluated the N372H (rs144848) common variant of BRCA2 and determined that this polymorphism was not associated with PFS or OS. However, given the critical role that BRCA2 plays in HR, the effect that germline mutation in BRCA1 or BRCA2 has on increasing a woman’s risk of breast and ovarian cancer and the fact that half the high-grade serous ovarian cancers have defects in HR (27, 44), other SNPs in BRCA1/BRCA2 may have clinical relevance. For example, extensive linkage disequilibrium (LD) exists across the BRCA1 gene with only two blocks of common genetic variations. The P871L polymorphism provided information regarding one LD block in the BRCA1 gene. Evaluation of a polymorphism in the other LD block in BRCA1 (e.g., Q356R) would be an efficient strategy for accessing the potential impact of all of the polymorphisms in the second LD block in the BRCA1 gene on treatment efficacy and/or outcome.

The clinical value of genetic variants in terms of prognosis and drug response are often more subtle than prognostic clinical factors making them more challenging to evaluate in cancer patients (38). Broadly defined clinical groupings and heterogeneous treatment regimens can have a strong influence on outcomes and mask the effects of individual genetic variants. One of the strengths of this study is that the population is relatively homogeneous. All of the women had optimally debulked stage III EOC and were uniformly managed as a result of their participation in a GOG phase III treatment protocol. Evaluation of other common polymorphisms in DNA repair remains a viable area of research given the role that DNA repair plays in drug sensitivity and resistance as well as genomic chaos and poor outcome in solid tumors like EOC (44) with high initial response rates to platinum and taxane-based chemotherapy and poor long term survival with 10-year survival rates approaching 10%.

In summary, common polymorphisms in BRCA1 (P871L) and BRCA2 (N372H) were not associated with PFS or OS in women with optimally resected, stage III EOC treated with C + P. This study provides supportive evidence that germline mutations in BRCA1 enhance sensitivity and PFS.

## Conflict of Interest Statement

The authors declare that the research was conducted in the absence of any commercial or financial relationships that could be construed as a potential conflict of interest.
